# Antinociceptive interaction of (±)-CPP and propentofylline in monoarthritic rats

**DOI:** 10.1186/ar4030

**Published:** 2012-08-24

**Authors:** Francisco Morales, Luis Constandil, Teresa Pelissier, Alejandro Hernández, Claudio Laurido

**Affiliations:** 1 Laboratory of Neurobiology, Department of Biology, Faculty of Chemistry and Biology, University of Santiago of Chile, Ave. Libertador B. O'Higgins 3363, Casilla 40 Correo 33, Santiago 917002, Chile; 2 Program of Molecular and Clinical Pharmacology, Institute of Biomedical Sciences (ICBM), Faculty of Medicine, University of Chile, Independencia 1027, P.O. Box 70000 Santiago 7, Santiago, Chile

## Abstract

**Introduction:**

Multiple studies have shown that glial cells of the spinal cord, such as astrocytes and microglia, have close contact with neurons, suggesting the term tripartite synapse. In these synapses, astrocytes surrounding neurons contribute to neuronal excitability and synaptic transmission, thereby increasing nociception and thus the persistence of chronic pain. Conversely, the *N*-methyl-D-aspartate (NMDA) receptor is crucial in the generation and maintenance of chronic pain. It has multiple sites of modulation. One is the site of recognition of extracellular neurotransmitter (glutamate), which can be blocked by competitive antagonists such as (3-(2-carboxipiperazin-4)1-propyl phosphonic acid), (±)-CPP, resulting in a blockade of the calcium current and thus the intracellular transduction process. In the present study, we investigated whether the potential antinociceptive effect of glial inhibition produced by propentofylline (PPF) can be enhanced when combined with an NMDA-receptor inhibitor such as (±)-CPP.

**Methods:**

We used Sprague-Dawley monoarthritic rats. The monoarthritis was induced by injection of complete Freund adjuvant in the right tibiotarsal joint. Four weeks later, rats were treated with PPF (1, 10, 30, and 100 μg/10 μl) intrathecally (i.t.) for 10 days, injected once with (±)-CPP (2.5, 5, 12.5, 25, 50, and 100 μg/10 μl, i.t.), or both treatments combined. The antinociceptive effect was evaluated on day 11 for PPF and immediately to (±)-CPP, by assessing the vocalization threshold to mechanical stimulation of the arthritic paw.

**Results:**

The data indicate that intrathecal administration of increasing concentrations of (±)-CPP or PPF produced a significant dose-dependent antinociceptive effect with respect to monoarthritic rats receiving saline. The linear regression analysis showed that the dose that produces 30% of maximal effect (ED_30_) for i.t. (±)-CPP was 3.97 μg, and 1.42 μg for i.t. PPF. The administration of the PPF and (±)-CPP combination in fixed proportions of ED_30 _produced a dose-dependent antinociceptive effect, showing an interaction of the supraadditive type.

**Conclusions:**

The results suggest that glia inhibitors can synergically potentiate the effect of glutamate blockers for the treatment of chronic inflammatory pain.

## Introduction

Pain is a sensory modality that, in its acute form, performs the physiological role of alerting the individual of real or potential tissue damage. It is the immediate consequence of the pain-pathways activation (nociceptive system), ongoing in a temporal fashion, and usually resolves when the painful stimulus is removed. Conversely, when pain lasts, even after the lesion has been healed, or when pain is originated without apparent tissue damage and lasts for more than 6 months, it is considered pathologic and called chronic pain [1].

The information collected by the nociceptors is driven by primary afferent fibers to the spinal cord where they synapse and transmit nociceptive information to projection neurons located in the dorsal horn of the spinal cord. These projection neurons relay the information to supraspinal centers through ascending pathways. The first-order neurons release a number of neurotransmitters, among others, glutamate and substance P. Substance P stimulates NK-1 receptors that produce a slow and prolonged depolarization in the projection neuron. Glutamate binds to AMPA receptors, increasing depolarization. When nociceptive stimulation frequency is greater, it generates a membrane depolarization that triggers the release of ion Mg^2+ ^from the NMDA receptor [2], promoting the entry of Ca^2+ ^and the subsequent activation of the enzyme nitric oxide synthase, generating nitric oxide production (NO). NO is a gas that diffuses rapidly through the cell membrane and acts as an excitatory retrograde messenger in the neurons that generate it, as in the presynaptic elements and adjacent astrocytes. This event, classified as positive feedback, has an important role in the development of synaptic neuroplasticity mechanisms, as has been shown for hippocampal LTP [3] and spinal potentiation known as spinal cord windup, generated against a high and low frequency of C-fiber stimulation, respectively. As a result, the perception of pain increases significantly, a potentiation phenomenon in the origin of the generation of chronic pain.

The NMDA receptors are tetramers [4] that can be assembled in different configurations. The NR1 subunit is essential for the functionality of the receptor, whereas the NR2 subunits determine the biophysiologic properties of the channel, like the conductance and the average time of opening or blocking sensitivity to Mg^2+ ^[5]. The cloning of the receptor subunits revealed that the NR1 subunit has a glycine-binding site, whereas the NR2 subunit has a glutamate-binding site, which can be blocked by competitive antagonists such as (±)-CPP, resulting in a blockade of the Ca^2+ ^current, and therefore the intracellular transduction process, as well as the inhibition of the windup phenomenon [6]. Moreover, a number of other NMDAR antagonists, such as ketamine and ifenprodil acting on different receptor sites, have been shown to present antinociceptive effects in models of inflammatory and neuropathic pain [7-11]. This indicates that the (±)-CPP could be used as an analgesic, because this receptor is involved in the induction and maintenance of central sensitization.

As mentioned earlier, the NMDA receptor is important in the establishment of chronic pain; however, today we know other factors that can modulate this pain, such as glial cells [12]. In the last decade, numerous studies have shown that glial cells of the spinal cord have a close communication with neurons, proposing the term tripartite synapse [13]. This synapse contributes to the modulation of neuronal excitability and synaptic transmission by increasing nociception and thus the persistence of chronic pain. It has been found that astrocytes and microglia in the dorsal horn of the spinal cord are active against a variety of conditions that cause chronic pain and hyperalgesia, such as subcutaneous swelling, subcutaneous administration of inactivated mycobacterium [14], and trauma peripheral nerve [15], among others [16].

Once activated glial cells release several neuroactive molecules capable of inducing or magnifying the pain, such as NO, prostaglandins, arachidonic acid, excitatory amino acids (glutamate, aspartate, cysteine), quinolinic acid, and growth factors, as well as variety of proinflammatory cytokines, such as interleukin-1β, interleukin-6, and tumor necrosis factor [17]. Glial cells and neurons have receptors for cytokines. It is accepted that cytokines have a role as neuromodulators in the central nervous system, specifically at the level of second-order nociceptive neurons. In this regard, it has been reported that IL-1β is able to increase the C-fiber response and windup activity in the spinal cord [18] at the level of nociceptive afferent terminals, where IL-1β increases the release of substance P and glutamate [19].

In this context, it is apparent that the main strategy to suppress the communication between glia and spinal neurons is through the possibility of pharmacologically disrupting the glial function. In this regard, different drugs have been identified that inhibit the activity of glia, including propentofylline (PPF) [20]. PPF has inhibitory effects on the activity of phosphodiesterase types I, II, and IV and on adenosine extracellular transporters in glial cells [21], thereby modifying intracellular cyclic nucleotide homeostasis, leading to a decrease of the production of proinflammatory cytokines and free radicals in these cells. This is supported by studies in which has been found an inhibition of the release of tumor necrosis factor and interleukin 1, as well as the formation of oxygen radicals, in microglia cultures activated by LPS treatment and subsequently challenged with PPF. Moreover, increased cAMP-dependent signaling has been shown to increase the expression of antiinflammatory cytokine IL-10 [22]. Therefore, PPF may increase the production of antiinflammatory cytokines and, in turn, downregulates the production of proinflammatory cytokines.

PPF also functions as a reuptake inhibitor of adenosine [23]. This is potentially important because adenosine has been proposed to play a role in neuropathic pain. Adenosine presynaptically inhibits the release of substance P and glutamate, and postsynaptically decreases the action of substance P and glutamate [24]. Inhibition of substance P and glutamate release can attenuate central sensitization and, consequently, could decrease pain.

Because NMDA receptors and glia have an important role in the pathophysiology of chronic pain, we propose to evaluate whether the coadministration of (±)-CPP and PPF could enhance the analgesic effect of each drug on chronic inflammatory pain, by using an isobolographic analysis. The ultimate goal of drug combination is to obtain effective analgesia with a reduction in the incidence and severity of side effects, which can be achieved by using lower doses of the drugs [25].

## Materials and methods

### Animals

In total, 152 male monoarthritic Sprague-Dawley rats (225 to 250 g) were used in this study. The experimental groups were constituted by six animals in each group. All animals were obtained from the facilities of the Faculty of Medicine of the University of Chile, held in a light-dark cycle of 12/12 hours, starting at 8:00 AM, food and water *ad libitum*. After each experiment, rats were killed by using an overdose of urethane (3 g/kg, intraperitoneal, i.p.)

The experiments were conducted in accordance with the "Guide for the Care and Use of Laboratory Animals of National Institutes of Health (NIH)" [26] and the rules of the International Association for the Study of Pain (IASP) "Models of animal pain and ethics in experimental animals" [27] and "Ethical standards in research and management of pain." Furthermore, the experimental protocols were approved by the Bioethics Committee of the Universidad de Santiago de Chile.

### Induction of monoarthritis

Monoarthritic rats were used as a model of chronic inflammatory pain. Monoarthritis was induced in rats of 120 to 150 g by the method described by Butler *et al.*, [28]. In brief, rats were inoculated with a volume of 50 μl of Freund adjuvant, in the right ankle joint. The adjuvant consisted of a solution of 60 mg of *Mycobacterium butiricum*, 6 ml of mineral oil, 4 ml of sodium chloride (0.9%), and 1 ml of Tween 80. Subsequently, this mixture was autoclaved at 120°C for 20 minutes and stored at room temperature until use. Before injection, the solution was homogenized by constant stirring. The injection of adjuvant produces a localized arthritic syndrome that becomes stable around the fourth week after inoculation, and establishes a persistent pain with hyperalgesia of the tibiotarsal joint, which is maintained for a period exceeding 2 months. Around 90% to 95% of the injected rats developed mechanical hyperalgesia. Monoarthritic rats were used between the fourth and the fifth weeks after induction of monoarthritis.

### Intrathecal injection

(±)-CPP (Tocris) was administered at single doses of 2.5, 7.5, 12.5, 25, 50, and 100 μg/10 μl. PPF (Sigma) was administered in repeated doses of 1, 10, 30, and 100 μg/10 μl, once daily for a period of 10 days. The two drugs were administered via i.t. injection in a volume of 10 μl and dissolved in saline; i.t. injection consists of administering the drug into the subarachnoid space between lumbar vertebrae L5 and L6 [29], by using a Hamilton syringe with a needle 26G × 1/2 inch'. The access to the subarachnoid space is evidenced by a slight movement in the tail of the rat as a result of the needle mechanical stimulation penetrating the meninges of the spinal cord. The daily PPF i.t. injection was done under brief halothane anesthesia (2 minutes).

### Experimental groups

To evaluate the antinociceptive effect of both drugs individually on monoarthritic rats, the vocalization threshold to mechanical stimulation (Randall-Selitto test) was used. The animals were separated in a first stage of experimentation into two groups: (a) intrathecal administration of (±)-CPP: 2.5, 7.5, 12.5, 25, 50, or 100 μg/10 μl (*n *= 6 for each dose); and (b) daily i.t. administration of increasing PPF concentrations of 1, 10, 30, or 100 μg/10 μl (*n *= 8 for each dose) for 10 days.

To evaluate the antinociceptive effect of the PPF and (±)-CPP combination, we conducted a second series of experiments. Both drugs were diluted in decreasing doses (1/3, 1/10, and 1/100) in relation to its ED_30_. Five groups were used:

1. Daily administration of ED_30 _of PPF i.t. for 10 days. At day 11, an i.t. injection of ED_30 _of (±)-CPP was done (*n *= 6).

2. Daily administration of ED_30 _of PPF i.t. for 10 days. At day 11, an i.t. injection of 1/3 of ED_30 _of (±)-CPP was done (*n *= 6).

3. Daily administration of ED_30 _of PPF i.t. for 10 days. At day 11, an i.t. injection of 1/10 of ED_30 _of (±)-CPP was done (*n *= 6).

4. Daily administration of ED_30 _of PPF i.t. for 10 days. At day 11, an i.t. injection of 1/30 of ED_30 _of (±)-CPP was done (*n *= 6).

5. Daily administration of ED_30 _of PPF i.t. for 10 days. At day 11, an i.t. injection of 1/100 of the ED_30 _of (±)-CPP was done (*n *= 6).

Controls were provided by normal and monoarthritic rats receiving saline, as follows:

1. Normal group of the same age of monoarthritic rats, receiving i.t. injection of saline before testing (*n *= 6).

2. Monoarthritic saline group, pooled from saline controls for the (±)-CPP, PPF, and combined (±)-CPP/PPF series, receiving i.t. daily injection of saline for a period of 10 days, followed by an i.t. injection of saline at day 11, or a single injection at day 11 (*n *= 16). The three groups were pooled because they showed no significant differences in vocalization threshold between them at any time of testing.

### Mechanical hyperalgesia

This behavioral test consists of adding a continuous and increasing pressure with a taper ending in blunt tip on the posterior knee joint of the rat to generate a nociceptive behavior. The response is evidenced by a vocalization or withdrawal reflex of the limb in response to stimulation. The pressure on the joint is increased gradually (linearly) up to 570 g, a value that does not harm the animal. The equipment used for this test was called analgesiometer Ugo Basile. Each animal was tested 2 times at 5, 15, 30, and 60 min for monoarthritic rats treated with (±)-CPP or the combination of PPF and (±)-CPP, and at 15, 30, and 60 min for monoarthritic PPF-treated rats. After the experiment, all rats were killed with an overdose of urethane. Grams of pressure, which expresses rat nociceptive behavior, were saved for later analysis. The data were expressed as percentage change to baseline and were then averaged over the different groups and different times. Later, the area under the curve (AUC) was calculated, by using the Microcal Origin V 6.0 program, and the groups were compared statistically.

### Isobolographic analysis

The evaluation of the interaction between both drugs was performed by using isobolographic analysis [25]. The isobologram is a graphic method that consists of calculating the theoretic additive dose for each level of effect and their statistical comparison with the combination dose that produces the same effect experimentally. Equieffective doses of both drugs alone are needed to calculate the expected dose in a combination. To this end, we determined the dose that produces 30% of maximal effect (ED_30_) by using a linear regression analysis from the dose-response curve of six increasing doses of (±)-CPP and the previously mentioned for increasing doses of PPF. Once we obtained the ED_30 _of both drugs, a graph was constructed by placing in the y-axis of the ED_30 _point of (±)-CPP and the x-axis point of the ED_30 _of PPF. The union of two points by a straight line (isobolo), also known as a line of additivity or no interaction, helped to establish the type of interaction (synergism or antagonism) of both compounds. The interaction between both drugs was carried out by an administration of 1, 1/3, 1/10, 1/30, and 1/100 of the ED_30 _(±)-CPP, and PPF. The coadministration was performed through intrathecal PPF ED_30 _daily for 10 days. The antinociception was assessed on day 11 with the Randall-Selitto test and then followed by i.t. administration of ED_30 _(±)-CPP; antinociception was assessed by the same test. Then the ED_30 _of the association of both drugs (ED_30 _experimental), from a dose-response curve, was obtained by linear regression analysis. This dose was compared statistically with the dose that theoretically represents the simple addition of effects, obtained by the following formula:

$$\text{E}{\text{D}}_{30}\text{ theoretical additivity}=\text{E}{\text{D}}_{30}\text{ PPF/}\left(\text{P}1+{\text{R}}^{*}\text{P}2\right)$$

Where R is the power ratio between the two drugs given alone, P1 is the proportion of the drug (PPF) in the mixture, and P2 is the proportion of drug 2 ((±)-CPP) in the mixture.

The graphic region in which is located the experimental value (ED_30 _experimental) in relation to the theoretic value (ED_30 _theoretic additivity) determines the type of interaction: If the value is located under the line of additivity and is statistically different from the theoretic value, the type of interaction is synergistic or supraadditive (effect greater than the sum of the individual effects of drugs); if located next to the line of additivity and not statistically different from the theoretic value, the interaction is simple additivity (equal effect of the sum of each drug); conversely, if the experimental value lies above the line of additivity and is statistically different from the theoretic nature of the interaction, it is subadditive or antagonistic. At the same time, we calculated the interaction index (I.I.) between the drugs, obtained from the following formula:

$$\text{I}\text{.I}\text{. = E}{\text{D}}_{30}\text{ experimental/E}{\text{D}}_{30}\text{ theoretic additivity}$$

This index, when less than 1 corresponds to a synergistic interaction, when equal to 1, corresponds to an additive interaction, and when greater than 1 is an antagonistic interaction [30].

### Statistical analysis

The results were expressed as mean percentage of antinociceptive effect ± standard error of the mean (SEM) for each experimental group, from baseline obtained before the injection of saline or each of the drugs under study, as appropriate. The quantification of the antinociceptive effect (%AE) of the drugs tested were calculated as a percentage change in AUC from baseline (basal) for each rat, and set a maximum pressure cut-off of 570 g in the Randall-Selitto, according to the following formula:

(1) $$\text{AU}{\text{C}}_{\text{post}}-\text{AU}{\text{C}}_{\text{pre}}=\text{AU}{\text{C}}_{\text{drug effect}}$$

(2) $$\%\;\text{AE}=\left(\text{AU}{\text{C}}_{\text{drug effect}}/\text{AU}{\text{C}}_{\text{cut-off}}\right)\times 100,$$

Where AUC_pre _and AUC_post _are approximate integrals of the curves obtained by the method of trapezoids and pre-post drug injection, respectively, according to Eq. 1. The AUC_drug effect _values are the integrals of the real effect of the drug. The antinociceptive effect (AE) was calculated according to Eq. 2, where the AUC_cut-off _corresponds to the area of maximum pressure possible on the animal.

To analyze the time-course of the antinociceptive effect of increasing doses of i.t. (±)-CPP and PPF, two-way ANOVA was performed. It allowed us to assess both intergroup comparisons (vocalization-threshold changes under different treatments) and intragroup comparisons (vocalization thresholds along the time), followed by the Bonferroni multiple comparisons test. To analyze the percentage antinociception obtained from the area under the time-course curves, one-way ANOVA was used, followed by Tukey-Kramer multiple comparisons test. To assess differences for the theoretic ED_30 _and experimental ED_30_, the two-tailed Student *t *test was used. All statistical analyses were performed with the Prism 3.0 software (GraphPad Software, Inc., San Diego CA, USA).

## Results

### Dose-response of (±)-CPP on mechanical nociception in monoarthritic rats

**Figure 1:**
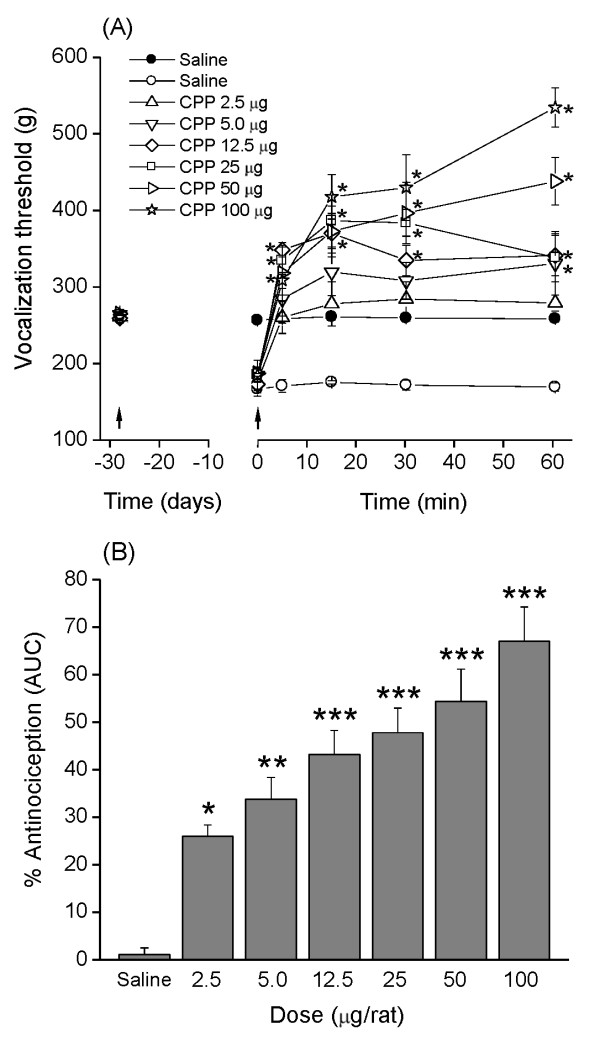
**Antinociceptive effect of (±)-CPP in monoarthritic rats**. **(A) **Time-course of the antinociceptive effect of increasing doses of i.t. (±)-CPP (2.5, 5.0, 12.5, 25, 50, and 100 μg/rat). Vocalization thresholds were measured before (left arrow), and then 28 days after monoarthritis induction, and after a single injection of CPP. Open symbols, values from monoarthritic rats. Solid symbols, values from normal rats receiving saline under a similar protocol. The right arrow corresponds to CPP or saline injection. Values are expressed as mean ± standard error of the mean (SEM); *n *= 6 rats per group. Two-way ANOVA indicates a significant effect for the (±)-CPP Treatment factor (*F*_(6, 175) _= 39.32; ANOVA *P *< 0.0001), as well as for the Time factor (*F*_(5, 175) _= 56.64; ANOVA *P *< 0.0001). Bonferroni multiple comparisons post hoc test showed that vocalization thresholds of all (±)-CPP treated rats (2.5, 5.0, 12.5, 25, 50, and 100 μg/rat) were significantly higher (p < 0.05) than the corresponding threshold of saline-treated animals (symbols omitted). In addition, Bonferroni multiple comparisons *post hoc *test showed that vocalization thresholds of rats after receiving the four highest doses of (±)-CPP were significantly higher (*P < 0.05) than the threshold measured before monoarthritis induction. **(B) **Ordinate indicates percentage antinociception obtained from the area under the time-course curves from (A) (see Materials and methods). Data are expressed as mean ± standard error of the mean (SEM), and were analyzed by using one-way ANOVA followed by Tukey-Kramer multiple comparisons test (*P < 0.05; **P < 0.01; ***P < 0.001; compared with monoarthritic rats receiving saline).

The administration of (±)-CPP (2.5, 5, 12.5, 25, 50, or 100 μg/10 μl) increased the vocalization threshold measured at 5, 15, 30, and 60 min after injection compared with rats receiving saline (Figure 1A), well above the pre-monoarthritis threshold. Areas under curves indicate that rats administered with saline showed a percentage of antinociception of 1.1% ± 1.4%, whereas rats administered with increasing doses of (±)-CPP showed a percentage of antinociception of 26.0% ± 2.4%, 33.9% ± 4.5%, 43.2% ± 5.0%, 47.8% ± 5.2%, 54.4% ± 6.8%, and 67.0% ± 6.8%, respectively (Figure 1B). In all cases, they were significantly higher than the percentages represented by the saline, showing a dose-dependent increase in trend. The linear regression analysis of the percentage AE showed that the ED_30 _was 3.97 μg, with a 95% confidence interval (95% CI) of 2.35 to 6.7 μg.

### Dose-response of PPF on mechanical nociception in monoarthritic rats

Unlike the study with (±)-CPP, the PPF was administered over a longer term (that is, once daily for 10 consecutive days) to ensure that the glia became inactive. At day 11 of saline or PPF treatment, the animals were challenged with a single dose of saline (10 μl) and studied at 0, 15, 30, and 60 minutes after injection. The effect of PPF was evaluated by comparing the treatments as independent groups.

**Figure 2:**
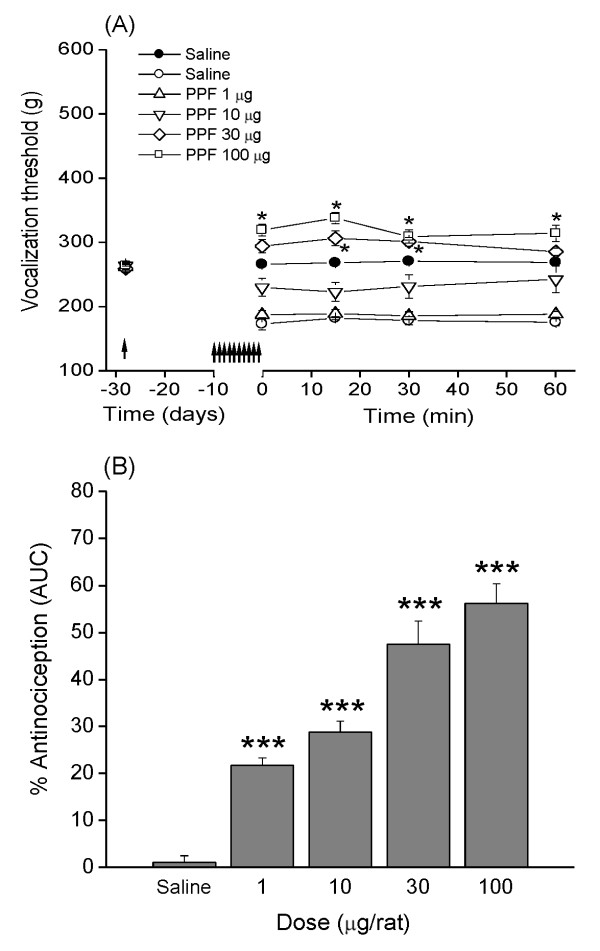
**Antinociceptive effect of PPF in monoarthritic rats**. **(A) **Time course of the antinociceptive effect of daily repeated injections of PPF in monoarthritic rats. Vocalization thresholds were measured before (left arrow), and then 28 days after monoarthritis induction, and 10 days after repeated injection of PPF (right arrows) (Time 0). Empty symbols represent values from monoarthritic rats. Solid symbols represent values from normal rats receiving saline under a similar protocol. Values are expressed as mean ± standard error of the mean (SEM); *n *= 6 rats per group. Two-way ANOVA indicates a significant effect for the PPF treatment factor (*F*_(4, 125) _= 132.20; ANOVA *P *< 0.0001) as well as for the time factor (*F*_(4, 125) _= 7.55; ANOVA *P *< 0.0001). Bonferroni multiple comparisons *post hoc *test showed that vocalization thresholds of rats receiving the three highest doses of PPF (10, 30, and 100 μg/rat) were significantly higher (*P *< 0.05) than the corresponding thresholds of saline-treated animals (symbols omitted). In addition, Bonferroni multiple comparisons *post hoc *test showed that vocalization thresholds of rats after receiving the highest doses of PPF were significantly higher (*P < 0.05) than the threshold measured before monoarthritis induction. **(B) **Ordinate indicates percentage antinociception obtained from the area under the time-course curves from (A) (see Materials and methods). Data are expressed as mean ± standard error of the mean (SEM), and were analyzed by using one-way ANOVA followed by Tukey-Kramer multiple comparisons test (***P < 0.001, compared with monoarthritic rats receiving saline).

The administration of saline i.t. for 10 days in monoarthritic rats produced an average threshold of vocalization at zero time of 174 ± 9.2 g. After the injection of saline challenge, this vocalization threshold was unchanged at 0, 15, 30, and 60 minutes after injection (Figure 2A). In the groups treated with 1, 10, 30, and 100 μg/10 μl PPF for 10 days, the vocalization threshold at 0 time was 183 ± 6.3, 226 ± 13.5, 288 ± 10.0, and 310 ± 8.8 g, respectively, which remained without modifications during the 60 minutes of measurement. These data show that PPF produced dose-dependent increases in the vocalization threshold in monoarthritic rats, the two higher doses raising the threshold above those observed in the premonoarthritis condition.

Area under curves indicates that monoarthritic rats injected with increasing doses of PPF (1, 10, 30, or 100 μg/10 μl) showed a percentage of antinociception of 32.8% ± 1.0%, 39.4% ± 2.4%, 50.1% ± 1.8%, and 54.4% ± 1.6%, respectively (Figure 2B), which were significantly higher than that observed in saline controls. Linear regression analysis allowed calculation of an ED_30 _of 1.42 μg with a 95% CI of 0.88 to 2.27 μg.

### Dose-response of the combination of PPF and (±)-CPP: isobolographic study

In a second series of experiments, (±)-CPP and PPF were administered together in a proportion obtained from their respective ED_30_, which made possible to calculate the theoretic additive dose, generating a series of theoretic doses shown in Table 1.

**Table 1:** Fixed proportions, equieffective and theoretically additive, used for the combination of both drugs

Fixed proportions	*n*	Equieffective dose (μg)		Theoretically additive (μg)
		
		PPF	(±)-CPP	PPF + (±)-CPP
1	6	1.42	3.97	5.39
1/3	6	0.47	1.32	1.79
1/10	6	0.14	0.4	0.54
1/30	6	0.05	0.13	0.18
1/100	6	0.01	0.04	0.05

*N*, number of rats.

**Figure 3:**
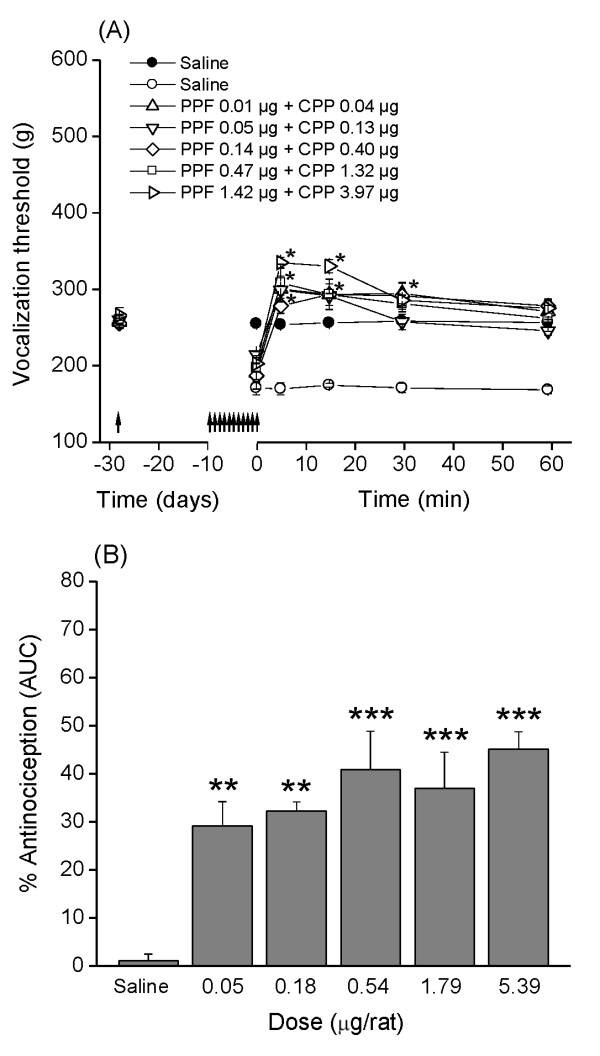
**Antinociceptive effect of PPF/(±)-CPP combination in monoarthritic rats**. **(A) **Time course of the antinociceptive effect of daily repeated injections of PFF followed by a single injection of (±)-CPP in monoarthritic rats. Vocalization thresholds were measured before (left arrow) and then 28 days after monoarthritis induction, and after 10 repeated and a single injection of PPF and (±)-CPP, respectively (right arrows) (Time 0). Open symbols represent values from monoarthritic rats. Solid symbols represent values from normal rats receiving saline under a similar protocol. Values are expressed as mean ± standard error of the mean (SEM); *n *= 6 rats per group. Two-way ANOVA indicates a significant effect for the PPF/(±)-CPP treatment factor (*F*_(5, 180) _= 51,78; ANOVA *P *< 0.0001) as well as for the time factor (*F*_(5, 180) _= 44.37; ANOVA *P *< 0.0001). Bonferroni multiple comparisons *post hoc *test showed that vocalization thresholds of all PPF/(±)-CPP treated rats were significantly higher (*P *< 0.05) than the corresponding threshold of saline-treated animals (symbols omitted). In addition, Bonferroni multiple-comparisons *post hoc *test showed that vocalization thresholds of rats after receiving the highest doses of PPF/(±)-CPP were significantly higher (*P < 0.05) than the threshold measured before monoarthritis induction. **(B) **Ordinate indicates percentage antinociception obtained from the area under the time-course curves from (A) (see Materials and methods). Data are expressed as mean ± standard error of the mean (SEM), and were analyzed by using one-way ANOVA followed by Tukey-Kramer multiple comparisons test (**P < 0.01; ***P < 0.001; compared with monoarthritic rats receiving saline).

In the five groups treated with increasing doses of the PPF/(±)-CPP combination (according to Table 1), the vocalization threshold increased for all doses, starting at 5 minutes, and remained elevated until 60 minutes after injection (Figure 3A). For the three higher doses of the combination, the vocalization threshold remained above the premonoarthritis threshold throughout the testing period.

The %AE (Figure 3B) indicates that monoarthritic rats injected with equieffective doses of the PPF/(±)-CPP combination showed a percentage of antinociception of 29.1% ± 5.0%, 32.1% ± 1.9%, 40.8% ± 7.9%, 36.9% ± 7.4%, and 45.0% ± 3.6%, which were significantly higher than that observed in saline controls. Linear regression analysis showed that the ED_30 _for the PPF/(±)-CPP combination was 0.063 μg with a 95% CI of 0.012 to 0.334 μg.

**Figure 4:**
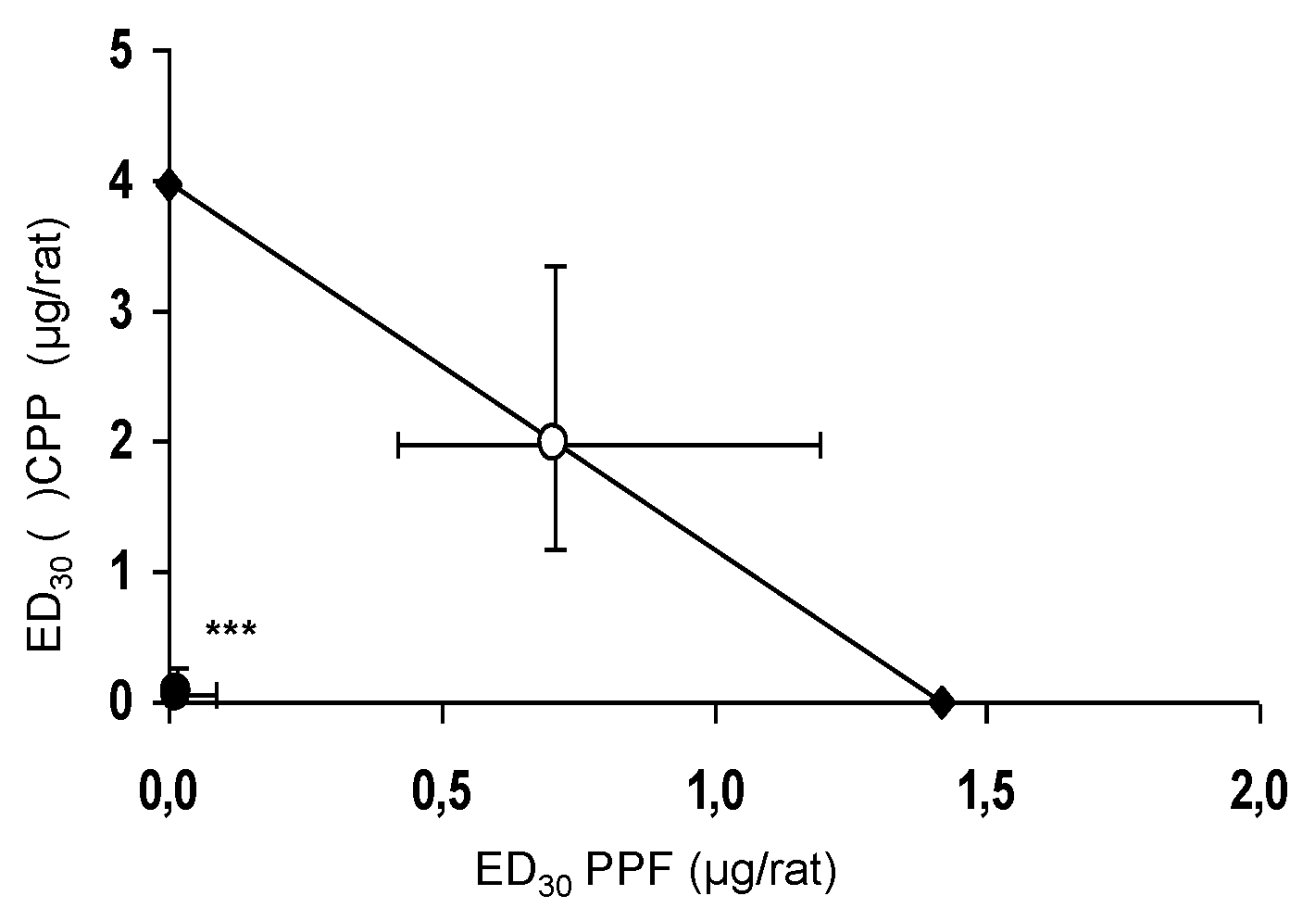
**Isobologram for the coadministration of PPF and (±)-CPP at fixed-ratio combinations**. The white circle on the straight line represents the point of theoretic activity calculated with a confidence limit of 95%, whereas the black circle under the straight line corresponds to the experimental point obtained with the monoarthritic rats (95% confidence limit). The experimental point was significantly different from the theoretically calculated point (mean ± SEM; ***P < 0.001, two-tailed Student *t *test), indicating supraadditive synergy in the Randall-Selitto test.

The combined effect of both drugs was analyzed by constructing an isobologram graph (Figure 4), which shows that the antinociceptive activity induced by coadministration of fixed proportions of the ED_30 _for PPF and (±)-CPP produced a greater antinociceptive effect than a simple additivity in monoarthritic rats. This result is achieved because the ED_30 _point for the combination is under the curve of isobolo and statistically different from the ED_30 _theoretically additive, indicating that the effect of the combination of both drugs is supraadditive (*t *= 2.879; *P *< 0.001, two-tailed Student *t *test). The interaction index between PPF and (±)-CPP was 0.024.

## Discussion

The results of this study show that the analgesic effect observed by combining PPF (a glial cells inhibitor) and (±)-CPP (an NMDA-receptor antagonist) on the paw-pressure test is supraadditive, in rats with chronic inflammatory pain. The ED_30 _obtained for (±)-CPP was 3.97 μg, and for PPF, 1.42 μg, whereas the ED_30 _of the combination was 0.063 μg, which was significantly lower than that expected by simple additivity. The ED_50 _was not used because the maximum effect of the drugs administered separately did not exceed 60% of the maximum effect.

As pointed out elsewhere [31-34], a supraadditive effect of combining two drugs producing the same effect could occur only if the mechanisms of action involved are totally or partially different (that is, "purely mutually nonexclusive" or "partially or nonpurely nonexclusive," as defined by Chou [31]), but not when the mechanism of action is the same for the two combined drugs. In the case of combining PPF and (±)-CPP, the mechanisms of action are partially independent and therefore consistent with the supraadditive effect found in the present study.

Some evidence supports that the administration of PPF to cultures of microglia from neonatal rat brain, activated by lipopolysaccharides, inhibits secretion of tumor necrosis factor (TNF-α), interleukin 1 (IL-1), and oxygen radicals [21]. Similar results obtained from microdialysis in the lumbar spinal cord of rats submitted to sciatic nerve chronic constriction injury have been reported [35]. It seems that inhibition by PPF of glial proinflammatory cytokine secretion is mediated by the cAMP-PKA pathway, because PPF effects are mimicked by dibutyryl-cAMP [36], and cAMP-PKA signaling represses proinflammatory cytokine gene expression in microglia [37]. However, the mechanisms of action of PPF are not yet clear. For instance, PPF has been shown to reinstate the decreased expression of glutamate transporters GLT-1 and GLAST produced for the L5 nerve transection in mice [38], thus promoting glial glutamate uptake and thereby glutamate excitotoxicity, therefore decreasing nociception by a mechanism different from proinflammatory cytokine repression. Furthermore, it has been reported that PPF decreases hyperalgesia induced by intracisternal BDNF administration [39], which may constitute another different mechanism from the previously mentioned. BDNF synthesis is increased not only in primary afferents during chronic pain [40,41] but also in second-order nociceptive neurons [42,43] and glial cells [44,45] of the dorsal horn. It has been claimed that BDNF promotes pain through two different mechanisms: (a) by potentiating the glutamatergic transmission in the spinal cord via increased glutamate release and enhanced synaptic efficacy at the postsynaptic level [46], and (b) by reducing the expression of the KCC2 transporter in dorsal horn neurons, which leads to a shift in the transmembrane anion gradient that causes normally inhibitory anionic synaptic currents to be excitatory; this latter mechanisms has been reported to be triggered only by glial-derived BDNF neurotrophin [44]. Because expression of the KCC2 transporter was found to be significantly reduced in spinal cord slices of rats with chronic inflammatory pain [47], it is likely that in the present study, PPF could reduce hyperalgesia by depressing glial BDNF release, thereby restoring the normal transmembrane anion gradient.

Conversely, it is accepted that the NMDA receptor is crucial in the transfer of nociceptive information in the spinal cord, specifically between the first and second nociceptive projection neurons [48]. Studies using antagonists of NMDA receptors have demonstrated their effectiveness as antinociceptive drugs in animal models of central hypersensitivity induced by cutaneous application of the chemical irritant mustard oil, tested with brief electrical stimulation of the sural nerve and challenged with MK-801 and (±)-CPP [49]. For example, MK-801 (an uncompetitive antagonist of the NMDA receptor) prevents skin and tactile hyperalgesia induced by muscle noxious C-fiber stimuli [7,50], and (±)-CPP (a competitive antagonist of the glutamate-binding site on the NMDA receptor) specifically blocks the action of glutamate, thus producing analgesia in different pain models [12,51]. In the present study, we demonstrated that increasing doses of (±)-CPP have a dose-dependent antinociceptive effects in monoarthritic rats.

Thus, it seems clear that PPF and (±)-CPP act through different mechanisms, but it is also clear that PPF and (±)-CPP can functionally interact because PPF lowers glial release of BDNF, thus avoiding the potentiating effect of the glial-derived BDNF on the glutamatergic transmission in the spinal cord. Therefore, the antihyperalgesic mechanisms of action of PPF and (±)-CPP are only partially independent, because PPF- and (±)-CPP-dependent effects can converge at the NMDA-receptor functionality, thus supporting supraadditive interactions when combined in equieffective doses.

## Conclusions

We showed for the first time that the glial inhibitor PPF can synergistically potentiate the effect of (±)-CPP, a drug that inhibits NMDA-receptor activity, thus opening the field of associating glial inhibitors to NMDA-receptor blockers in the pharmacologic treatment of chronic inflammatory pain. Glial inhibitors [52,53] and NMDA antagonists [54,55] have been associated with opioid therapy in a variety of painful conditions, but glial inhibitors and NMDA antagonists have not still assayed in combination clinical studies.

## Abbreviations

ANOVA: analysis of variance; AUC: area under curve; (±)-CPP: 3-(2-carboxipiperazin-4)1-propyl phosphonic acid; IL-1β: interleukin-1beta; NO: nitric oxide; PPF: propentofylline; TNF-α: tumor necrosis factor-alpha.

## Competing interests

The authors declare that they have no competing interests.

## Authors' contributions

FM, TP, and CL performed most of the experiments. TP performed experiments in inducing monoarthritis. LC, TP, AH, and CL conceived the study and participated in the design, in the interpretation of results, and in drafting the manuscript. All authors read and approved the final manuscript.
